# The mysterious diadenosine tetraphosphate (AP4A)

**DOI:** 10.1093/femsml/uqad016

**Published:** 2023-04-24

**Authors:** Victor Zegarra, Christopher-Nils Mais, Johannes Freitag, Gert Bange

**Affiliations:** Department of Chemistry and Center for Synthetic Microbiology, Philipps University Marburg, Marburg 35043, Germany; Department of Chemistry and Center for Synthetic Microbiology, Philipps University Marburg, Marburg 35043, Germany; Department of Biology, Philipps University Marburg, Marburg 35043, Germany; Department of Chemistry and Center for Synthetic Microbiology, Philipps University Marburg, Marburg 35043, Germany; Max Planck Institute for Terrestrial Microbiology, Marburg 35043, Germany

**Keywords:** dinucleoside polyphosphates, AP4A, heat shock, oxidative stress, nucleotide signaling, alarmones

## Abstract

Dinucleoside polyphosphates, a class of nucleotides found amongst all the Trees of Life, have been gathering a lot of attention in the past decades due to their putative role as cellular alarmones. In particular, diadenosine tetraphosphate (AP4A) has been widely studied in bacteria facing various environmental challenges and has been proposed to be important for ensuring cellular survivability through harsh conditions. Here, we discuss the current understanding of AP4A synthesis and degradation, protein targets, their molecular structure where possible, and insights into the molecular mechanisms of AP4A action and its physiological consequences. Lastly, we will briefly touch on what is known with regards to AP4A beyond the bacterial kingdom, given its increasing appearance in the eukaryotic world. Altogether, the notion that AP4A is a conserved second messenger in organisms ranging from bacteria to humans and is able to signal and modulate cellular stress regulation seems promising.

## Teaser

Diadenosine tetraphosphate (AP4A) is a nucleotide second messenger capable of reshaping cell metabolism by targeting specific biological pathways in bacteria.

## Origin, synthesis, and degradation of AP4A in bacteria

**Figure 1.**
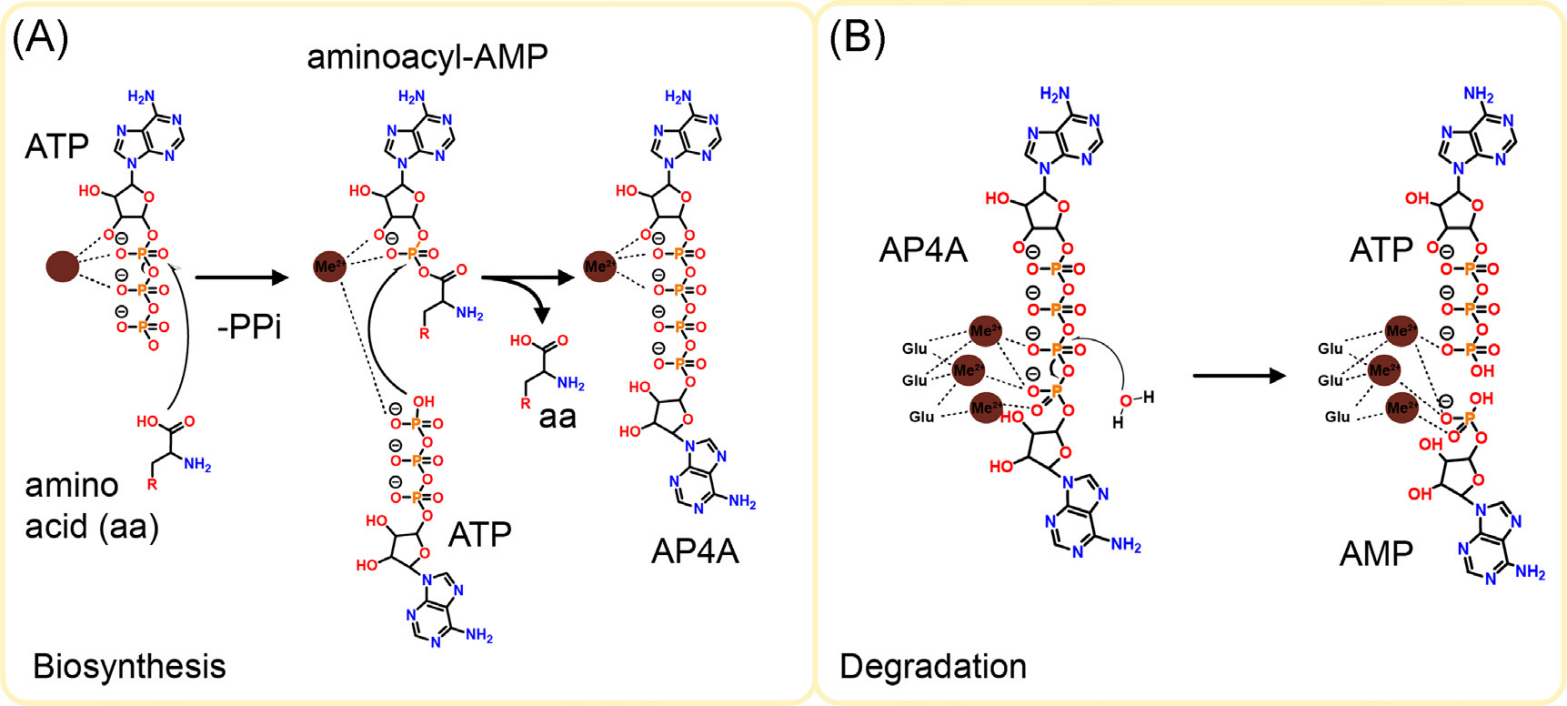
Mechanisms of AP4A synthesis and degradation. **(A)** Aminoacyl-tRNA synthetases produce AP4A in two concerted SN2 reactions, which are dependent on a divalent cation (brown circle). **(B)** Widespread and versatile Nudix hydrolases degrade AP4A. The *Escherichia coli* Nudix hydrolase RppH harbors three Mg^2+^ ions, catalyzing the reaction by selective activation of the *γ*-phosphate.

**Figure 2.**
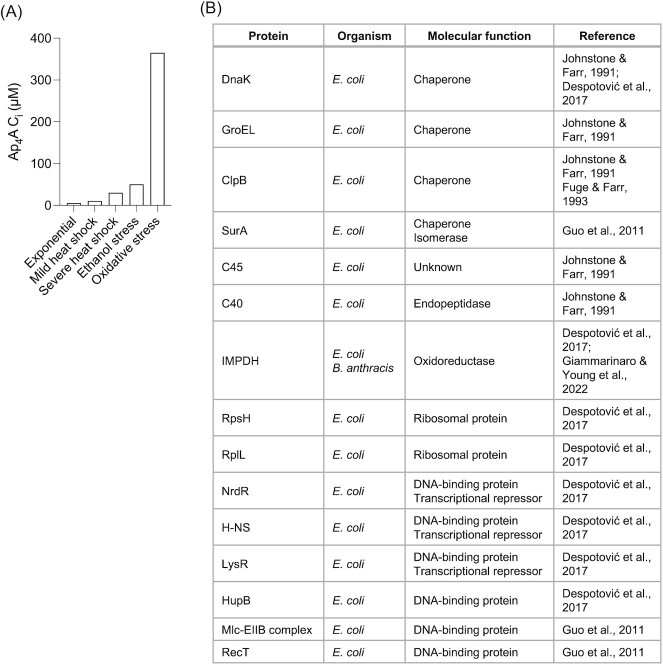
Physiology of AP4A in bacteria. **(A)** Intracellular levels of AP4A. These data were retrieved from *in vivo* studies in *E. coli* (exponential condition) (Farr et al. 1989, Despotović et al. 2017) and *Salmonella typhimurium* (all conditions) (Lee et al. 1983, Bochner et al. 1984). **(B)** Reported AP4A binders.

Dinucleoside polyphosphates (NP_*n*_N; where N represents adenosine, guanosine, uridine, or cytidine and *n* refers to the number of phosphates) are a class of nucleotides found in all kingdoms of life (Kisselev et al. 1998). In bacteria, the most abundant dinucleoside tetraphosphates (NP4As) are GP4A, CP4A, UP4A, and AP4A (Costes et al. 1987), the latter being the focus of this review. Initially discovered in 1966 (P. G. Zamecnik et al. 1966), diadenosine tetraphosphate (AP4A) is composed of two adenosine moieties linked by a polyphosphate chain of four phosphates connected by phosphoester bonds to the corresponding 5′-hydroxyl groups (Fig. 1A). Increased levels of AP4A have been consistently identified in bacteria in response to a wide variety of stresses, in particular temperature shift and oxidative damage (Costes et al. 1987, Plateau et al. 1987, Farr et al. 1989) (see below, Fig. 2A). Already early, these findings have led to the proposal that AP4A might act as a *bona fide* second messenger (e.g. P. Zamecnik 1983, Varshavsky 1983, Tshori et al. 2014). Besides that, the idea of AP4A being only a damage metabolite has also been discussed (Despotović et al. 2017). In the following sections, we will focus on our current knowledge of AP4A in bacteria.

### Biosynthesis of AP4A

Already in the middle of the past century, aminoacyl-tRNA synthetases (aaRSs) were recognized as a main source of AP4A, with lysyl-aaRS (Lys-aaRS) being the first example (P. G. Zamecnik et al. 1966). This class of enzymes is responsible for the specific esterification of an amino acid (aa) to the 3′-end of its cognate tRNA isoacceptor(s) (aa-tRNA). This central biological function is achieved through a two-step reaction: first, the enzyme activates the amino acid using Mg-ATP, resulting in the enzyme-bound aminoacyl-adenylate (aa-AMP) intermediate (Fig. 1A). Subsequently, the amino acid is transferred to either the 2′ or 3′ position of the 3′-terminal ribose of the cognate tRNA (Fig. 1A). However, when no cognate tRNA is bound, aaRS enzymes can employ ATP (instead of the tRNA) to split the aa-AMP into AP4A and the given aa (Fig. 1A) (Paul and Schimmel 2013).

Structural characterization of the seryl-aaRS from *Thermus thermophilus*, crystallized in apo, tRNA-, seryl-AMP-analogue, and AP4A-bound states, revealed several residues involved in this reaction (Biou et al. 1994). The base of the primary ATP is coordinated by an arginine-rich pocket conserved among Class II aaRSs and kept in place during both reaction steps. A dicationic metal ion, such as Mn^2+^ for SerS or Mg^2+^ or Zn^2+^ reported for other aaRSs, is coordinated in close proximity. These metals span up an octahedral coordination sphere that includes conserved glutamates as well as water molecules and the *α* and *β*-phosphate of the primary ATP (Cusack 1995, Swairjo and Schimmel 2005, Cain et al. 2019). In addition, a positively charged amino acid further coordinates the *α*-phosphate, activating ATP and stabilizing the intermediate aminoacyl-AMP by quenching the emerging charges. In close proximity to the bound ATP, a highly specific binding site for the cognate amino acid is located.

The positive polarization of the *α*-phosphate plays a crucial role during the first step of the reaction, in which the *α*-phosphate is targeted by the carboxyl moiety of the respective amino acid in a nucleophilic manner. While the new P-O-bond is established, inorganic pyrophosphate (PPi) is released. This S_N_2 reaction is driven by the stabilization of the intermediate in the active site and the irreversible release of PPi (Ozeir et al. 2019). For the second reaction step, an additional ATP is recruited to a second binding site in the absence of its cognate tRNA. Its *γ*-phosphate performs a nucleophilic attack on the *α*-phosphate of the primary ATP, aided by the positive polarization of the *α*-phosphate in the AA-AMP intermediate state.

Early studies of prokaryotic and eukaryotic aaRSs showed that the synthesis of AP4A was catalyzed by these enzymes with different efficiencies (Goerlich et al. 1982). AP4A production by a given aaRS requires the presence of its cognate amino acid, in the form of the aminoacyl-adenylate (aa-AMP) intermediate, within the active site of the enzyme (Fig. 1A). With respect to the catalytic ability to synthesize AP4A, it was suggested that aaRSs can be tentatively classified into groups with high (Phe, Lys, and His), low (Ile, Ser, Leu, Asp, Tyr, and Val), and undetectable (Arg, Trp) activities (Goerlich et al. 1982). While all aaRSs critically depend on magnesium as their divalent cation to chelate ATP, zinc has been recognized as a potent stimulator for Phe-aaRS (*E. coli*: 22-fold, *S. cerevisiae*: 35-fold, *Physarum polycephalum*: 20-fold) and Lys-aaRS to some extent (Plateau et al. 1981, Goerlich et al. 1982, Plateau and Blanquet 1982). Other aaRS enzymes (i.e. Tyr, Ile, Met, Ser, Val, and Trp) were insensitive to the stimulation of zinc (Goerlich et al. 1982). Thus, aaRS enzymes are a major source of AP4A production.

However, other adenylate-forming enzymes, which share the above-described two-step mechanism, produce AP4A from their respective substrates, AMP intermediates, and ATP. Examples include luciferase from firefly (*Photinus pyralis*) (Guranowski et al. 1990, ORTIZ et al. 1993), the acyl-coenzyme A synthetases from *Pseudomonas fragi* and *Saccharomyces cerevisiae* (Guranowski et al. 1994, Fontes et al. 1998), coumarate-CoA ligase from *Arabidopsis thaliana* (Pietrowska-Borek et al. 2003), DNA and RNA ligases (e.g. *Pyrococcus furiosus*, T4 phage) (Madrid et al. 1998, Atencia et al. 1999, Günther Sillero et al. 2002) and the adenylation domain of a non-ribosomal peptide synthetase from *Bacillus brevis* (Dieckmann et al. 2001). Moreover, propionate kinase from *S. typhimurium* has also been shown to form AP4A *in vitro*. Interestingly, the reaction catalyzed by this enzyme does not involve an AMP-intermediate, but the exact mechanism remains elusive to date (Simanshu et al. 2008a). A more comprehensive overview of NPXN synthesizing enzymes has been previously compiled (Fraga and Fontes 2011).

### Degradation of AP4A

Shared among all domains of life, AP4A is hydrolyzed by members of the versatile family of Nudix hydrolases (**nu**cleoside **di**phosphates linked to **x**) (Mildvan et al. 2005, McLennan 2006, 2013, Carreras-Puigvert et al. 2017). Members of this family possess an *α*-*β*-*α*-fold that harbors metal binding sites within the common Nudix motif GX_5_EX_7_REX_2_EEXG [I/L/V]. Typically, dicationic Nudix hydrolases degrade a wide range of substrates, often recognizing them with multiple nucleotide binding sites within direct proximity of the catalytic core (Swarbrick et al. 2005). In the case of AP4A, one or two adenosine binding sites orient the phosphate backbone close to the catalytically active metals, leading to a cleavage between the *γ*- and *δ*-phosphate (Fig. 1B; Levenson-Palmer et al. 2022). This asymmetrical hydrolysis results in the formation of AMP and ATP, which are bound with significantly lower affinities (Abdelghany et al. 2001, Hult and Berglund 2007).

In addition to the Nudix hydrolases discussed above, most organisms possess additional enzymes capable of symmetric AP4A degradation. In gram-negative bacteria, this reaction is carried out by homologus of the *E. coli* enzyme ApaH. Based on genetic analyses, the existence of this phosphatase family, commonly known as PPPs, has been suggested for other domains of life as well (Uhrig, Kerk et al. 2013, Uhrig, Labandera et al. 2013). Biochemical characterization of ApaH enzymes from Gram-negative bacteria such as *Myxococcus xanthus, Salmonella enterica*, and *E. coli* has revealed strictly symmetrical reaction mechanisms dependent on divalent cations like Mn^2+^ or Co^2+^ (Ismail et al. 2003, Sasaki et al. 2014). Despite lacking experimental structural data supported by biochemical characterization, a comparison of the well-characterized *M. xanthus* ApaH to the structure of the *Trypanosoma brucei* and *Shigella flexneri* homologus (PDB: 2QJC, 2DFJ) reveals the conserved metal binding site as well as a phosphate coordinated in direct proximity to these (Wang et al. 2006). Moreover, mutations in the metal-coordinating residues lead to a loss of hydrolase activity (Sasaki et al. 2014). The coordination between the ribose and the adenosine cannot be as easily deduced and would need further investigation.

For gram-positive bacteria, a recent study identified YqeK, a member of the HD domain superfamily, as an AP4A degrading enzyme (Minazzato et al. 2020). Genetic analysis revealed that the homologus of this enzyme can be found widely spread among the *Firmicutes, Thermotoga*, and *Thermus-Deinococcus* groups. Biochemical analysis has shown the degradation to be symmetrical with a substrate range including a variety of NPXN dinucleotides. Structures of YqeK homologus are available from *Streptococcus agalactiae* (PDB: 2OGI), *Bacillus halodurans* (PDB: 2O08), and *Clostridium acetobutylicum* (PDB: 3CCG). These reveal metal-binding sites for two Fe^3+^ cations as well as a nucleotide-binding site occupied by GDP (PDB: 2OGI) and dGDP (PDB: 2O08), respectively. This arrangement hints towards a metal-dependent cleavage mechanism between the *β*- and *γ*-phosphates of the substrate; however, biochemical evidence is still lacking. Taken together, a detailed mechanistic and structural characterization of the symmetrical cleavage of AP4A requires further attention.

## Physiology of AP4A in bacteria

Foundational studies performed in the 1980s revealed that in *S. typhimurium* and *E. coli*, the levels of AP4A increased in response to various physiological or environmental stresses (Plateau et al. 1987, Farr et al. 1989, Despotović et al. 2017). Despite no clear explanation of the function or mechanism of action of this and other Ap_*n*_Ns, these were crucial investigations that highlighted dinucleoside polyphosphates as potential stress alarmones. Almost 40 years later, efforts to understand the mechanistic and physiological relevance of AP4A are getting us closer to confirming these initial discoveries. The reported basal levels of AP4A in cells undergoing exponential growth fall between 0.2–1 µM in *E. coli* (Farr et al. 1989, Despotović et al. 2017) and ∼5 µM in *S. typhimurium* (Lee et al. 1983), two closely related gram-negative microorganisms (Fig. 2A). In exponentially growing *B. subtilis* cultures, higher levels of AP4A were detected at 24.2 µM (Giammarinaro and Young et al. 2022). When *S. typhimurium* faces a mild heat shock (28–42°C), the concentration of AP4A increases up to 10 µM and peaks at 30 µM upon severe heat shock treatment (28–50°C) (Lee et al. 1983). Moreover, if *S. typhimurium* is stressed with 10% ethanol, AP4A levels increase up to approximately 50 µM (Lee et al. 1983). To date, the highest concentration of AP4A in bacteria has been reported in *S. typhimurium* cells exposed to oxidative stress, reaching 365 µM (Bochner et al. 1984). Lastly, it was shown that in *E. coli*, a lethal concentration of kanamycin raises the levels of AP4A 20-fold, an effect that was even more pronounced when cells were challenged with hydrogen peroxide—two types of stress that coincide with the production of hydroxyl radicals (Ji et al. 2019). Interestingly, Δ*apaH* mutants exposed to kanamycin showed a 100-fold decreased survival compared to their WT counterparts, while a third cell strain overexpressing ApaH promoted tolerance against this antibiotic. On a side note, it has also been previously reported that Δ*apaH* is hypersensitive towards heat treatment (Johnstone and Farr 1991). This indicates that in the presence of aminoglycosides, an overaccumulation of AP4A induces cell death in *E. coli* (Ji et al. 2019). More broadly, all these data suggest that AP4A homeostasis might play an important role in stress tolerance in *E. coli*. Further studies are required to explain in detail the basis of these observations.

It was important to next decipher the relevance of elevated levels of AP4A in bacteria—is it merely a molecule that goes up upon stress, or is it physically interacting and regulating enzymatic functions to cope with said stress? In the light of the central and ubiquitous biochemical pathway by which AP4A is synthesized, the latter appears reasonable. Thus, several efforts were made to try and identify protein targets where AP4A would bind (Fig. 2B; Johnstone and Farr 1991, Fuge and Farr 1993, Guo et al. 2011, Azhar et al. 2014, Despotović et al. 2017, Giammarinaro and Young et al. 2022). In this sense, to our best knowledge, we summarized all the molecular structures reported to date on AP4A-bound proteins in Table 1. The targets for which a significant physiological effect was reported will be further described in this chapter. The first photo-crosslinking experiments performed in *E. coli* using radioactively labeled azido-AP4A revealed DnaK, GroEL, E90 (later identified as ClpB; Fuge and Farr 1993), C45, and C40 as direct targets of the nucleotide (Johnstone and Farr 1991). Due to previous data indicating that *dnaK* (Paek and Walker 1987) and *GroEL* (Fayet et al. 1989) mutants are hypersensitive to heat, similar to Δ*apaH* (Johnstone and Farr 1991), it was suggested that AP4A binding would preclude the ability of these enzymes to enhance thermal survival. Furthermore, it was observed that in heat-hypersensitive Δ*apaH* mutants, overexpression of ClpB would suppress thermosensitivity and promote survival. This hints at parallels to DnaK and GroEL and may explain the effects of AP4A accumulation upon heat stress (Fuge and Farr 1993).

**Table 1.** Overview of available structures with AP4A as a protein ligand. A search was performed in October 2022 with the AP4A acronym “B4P” as the search item at rcsb.org. The abbreviations are: aaRS—aminoacyl tRNA synthetase; IMPDH—inositol-monophosphate dehydrogenase; tbp—to be published.

Function	Protein	Organism	Function	PDB-ID	Citation
**Bacteria**					
**Producer**	Lys-aaRS	*Geobacillus stearothermophilus*	Synthesis of Lys-tRNAs	3A74	tbp
	LysU	*E. coli*	Synthesis of Lys-tRNAs	5YZX	tbp
	Propionate kinase TdcD	*S. typhimurium*	Anaerobic breakdown of L-threonine to propionate	2E1Z, 2E20	(Simanshu et al. 2008b)
**Degrader**	RppH	*E. coli*	AP4A hydrolysis and AP4A-decapping of mRNA	7SP3	(Levenson-Palmer et al. 2022)
	AP4A hydrolase (aq_158)	*Aquifex aeolicus Vf5*	AP4A degradation	3I7V	(Jeyakanthan et al. 2010)
**Target**	Pyrophosphatase (Family II PPase) activated by AP4A	*Clostridium perfringens*	Ppase activity is inhibited by AMP and activated by AP4A	3L2B	(Tuominen et al. 2010)
**Target**	IMPDH inhibited by AP4A	*B. subtilis*	NAD-dependent conversion of IMP to XMP	7OJ1	(Giammarinaro and Young et al. 2022)
**Target**	YxkO, role of AP4A unknown	*B. subtilis*	ADP/ATP-dependent NAD(P)H-hydrate dehydratation	3RQX	(Shumilin et al. 2012)
	Propionate kinase TdcD, role of AP4A unknown	*S. typhimurium*	Anaerobic breakdown of L-threonine to propionate	2E1Z, 2E20	(Simanshu et al. 2008b)
	tm0922, role of AP4A unknown	*Thermotoga maritima*	Unknown function	3RSF	(Shumilin et al. 2012)
**Eukaryotes**					
**Producer**	GLYRS	*Homo sapiens*	Gly-aaRS	2ZT5, 2ZXF	tbp
**Degrader**	Apa2	*Saccharomyces cerevisiae*	AP4A phosphorylase	4I5V	(Hou et al. 2013)
**Target**	HINT1 (histidine triad nucleotide-binding protein 1), AP4A inhibits HINT1	*Homo sapiens*	AP4A binds HINT1, disrupts interaction with the microphthalmia-associated transcription factor (MITF) → activates transcription of genes downstream of MITF in response to immunostimulation	6J65, 6J64	(Yu et al. 2019)
**Target**	NPP3 Ectonucleotide phosphodiesterase/pyrophosphatase-3, role of AP4A unknown	*Rattus norwegus*	membrane-bound glycoprotein that regulates extracellular levels of nucleotides	6F2Y	(Döhler et al. 2018)
**Target**	Cytosolic 5′-Nucleotidase II, role of AP4A unknown	*Homo sapiens*	dephosphorylation of 6-hydroxypurine nucleoside 5′-monophosphates	2XJC	(Walldén and Nordlund 2011)
**Target**	Adenosine Kinase, role of AP4A unknown	*Anopheles gambiae*	NTP-dependent phosphorylation of adenosine into AMP	3LOO	(Cassera et al. 2011)
**Target**	Adenylate kinases 1,2, role of AP4A unknown	*Homo sapiens*	2 ADP → ATP + AMP	2C9Y, 2C95	tbp

Later pulldown experiments employing biotin-labeled AP4A (Guo et al. 2011, Despotović et al. 2017) further extended the list of putative targets (Fig. 2B), of which we will focus on IMPDH, which was also found in *B. anthracis*, using radioactively labeled AP4A in a differential radial capillary action of ligand assay (DRaCALA) (Giammarinaro and Young et al. 2022). It was first thought that since other adenine dinucleotides could also bind and inhibit IMPDH function, AP4A could potentially do the same, but it wasn’t until recently that this was thoroughly explained (Giammarinaro and Young et al. 2022). Giammarinaro and Young et al. convincingly demonstrated that increased levels of AP4A dysregulate enzymatic activity by strongly binding (*in vitro* and *in vivo K*_d_s of 7.4–24.2 µM, respectively) into the CBS domains of two IMPDH subunits, inducing the assembly of IMPDH tetramers into less active octamers. Additional characterization of this interaction showed that the AP4A-bound IMPDH octamers are less active due to a less favorable change in the conformational geometry of their catalytic elements. Interestingly, it was further evidenced that inhibition of IMPDH disturbs the cellular nucleotide homeostasis by pushing the flux from IMP to GTP, a metabolic burden that decreased the survival of *B. subtilis* in response to heat shock stress.

An additional layer of NP4A-mediated (NP4As: AP4A, GP4A, CP4A, and UP4A) regulation identified in bacteria concerns the modulation of gene expression via the incorporation of alternative 5′-cap structures into nascent mRNAs during transcription initiation (Ferguson et al. 2020). Stresses that induce the accumulation of NP4As, particularly disulfide stress, lead to the incorporation of NP4A, at varying assimilation efficiencies, into mRNA as the initiating nucleotide during transcription. Moreover, the incorporation efficiency of NP4A nucleotides relative to ATP was significantly favored. Thus, NP4As can act as a protective 5′-modification that increases the transcript’s stability and possibly impacts gene expression (Luciano et al. 2019). This capping reaction is catalyzed by *E. coli*’s RNA polymerase and, at least *in vitro*, also by the lysyl-tRNA synthetase (Luciano and Belasco 2020). Intriguingly, ApaH, which also functions as the main NP4-decapping enzyme in proteobacteria, is inhibited by disulfide stress, raising the question of how the transcriptome can then accommodate to overcome this particular environmental stress. A recent discovery sheds light on this subject and reveals that during these NP4A-inducing stresses, RppH predominantly acts as the decapping enzyme for 5′-NP4A-capped mRNAs. Thus, RppH enables a cell to promptly reprogram its transcriptome in order to face the challenges ahead (Levenson-Palmer et al. 2022). All these data suggest that NP4As, with AP4A as the main player, are relevant regulators of bacterial metabolism and physiology.

## AP4A beyond the bacterial kingdom

Aside from aaRSs synthesizing AP4A, additional pathways for its synthesis have been reported in eukaryotes (for an extensive recollection, see Ferguson et al. 2020), further supporting the widespread and universal occurrence of these dinucleotides throughout diverse organisms. As such, the activation of ubiquitinin and ubiquitinin-like proteins, part of a post-translational modification system in eukaryotes, leads to the synthesis of AP3A and AP4A (Götz et al. 2019). In humans, the tumor suppressor proteins fragile histidine triad (FHIT) and Nudix hydrolase 2 (NUDT2) are responsible for the degradation of AP3A and AP4A, respectively (Barnes et al. 1996, McLennan 1999). The binding of AP4A–IMPDH appears to be conserved in eukaryotes (Fernández-Justel et al. 2019).

Similar to prokaryotes, the levels of AP4A increase upon exposure to various stresses, especially in response to oxidative, heat, or genotoxic stress (Brevet et al. 1985, Baltzinger et al. 1986, Baker and Ames 1988, Garrison et al. 1989, Guranowski 2004). This has been true for fungi, yeast, plants, *Drosophila*, and human cells. Quite strikingly, it was found that the pLysRS–AP4A pathway has profound implications in mast cell activation, reprogramming the cell from host defense to further enhance allergic diseases and cancer metastasis (Yannay-Cohen et al. 2009, Marriott et al. 2015, Yu et al. 2019, Govindaraj et al. 2021). Similarly, the LysRS-dependent production of AP4A can inhibit the activation of the stimulator of interferon genes (STING) pathway and attenuate inflammatory responses (Guerra et al. 2020). How complex this AP4A-dependent signaling pathway is and what the potential is for exploiting it for pharmacological and therapeutic reasons remains to be elucidated. Moreover, accumulation of AP4A has been detected in the eyes of glaucoma patients and in thrombocytes (Lüthje et al. 1987, Castany et al. 2011), rendering its accumulation likely to alter cellular fitness. Thus, it may be interesting to elucidate its role during degenerative processes such as aging and cellular differentiation.

Recently, the use of AP4A-based photoaffinity-labeling compounds made it possible to probe its interactome in human embryonic kidney cell lysates (Krüger et al. 2021). A total of 78 putative targets associated with RNA processing (mainly tRNA aminoacylation and mRNA splicing), carboxylic acid, and nucleotide metabolisms were identified. Interestingly, only 46% of these proteins were nucleotide binders (32% being ATP binders), meaning that AP3A and AP4A are not exclusively ATP competitors but also have other very distinctive, yet uncharacterized, roles. These data further support the idea that AP4A could be a relevant part of the regulatory inventory of all living organisms coping with stress. Both prokaryotes and eukaryotes can synthesize and degrade AP4A, which shows increased concentrations when coping with stress, and various AP4A-binding partners have been identified. However, characterization of the molecular mechanisms and consequences resulting from the binding of AP4A to each of its various putative binding partners in bacteria and eukaryotes is just at the beginning.
